# Mobile head CT with integrated radiation shielding reduces the need for additional personal protective equipment

**DOI:** 10.1093/bjr/tqag079

**Published:** 2026-04-11

**Authors:** Pierre Hillergren, Markus Hulthén, Robert Vorbau, Artur Omar

**Affiliations:** Medical Radiation Physics and Nuclear Medicine, Karolinska University Hospital, Stockholm 171 76, Sweden; Medical Radiation Physics and Nuclear Medicine, Karolinska University Hospital, Stockholm 171 76, Sweden; Medical Radiation Physics and Nuclear Medicine, Karolinska University Hospital, Stockholm 171 76, Sweden; Medical Radiation Physics and Nuclear Medicine, Karolinska University Hospital, Stockholm 171 76, Sweden; Department of Clinical Science, Intervention and Technology, Karolinska Institutet, Stockholm 171 76, Sweden

**Keywords:** mobile CT, intensive care, radiation protection, occupational exposure

## Abstract

**Objectives:**

To assess occupational exposure in a neurointensive care unit (NICU) equipped with a mobile head CT system.

**Methods:**

The exposure was investigated in a NICU equipped with the recently introduced Siemens Healthineers SOMATOM On.site mobile head CT system, which features integrated radiation shielding consisting of a foldable 0.5-mm lead curtain covering the front of the gantry opening and a detachable 1-mm lead radiation shield covering its back. Ambient dose (*H*^∗^(10)) and scattered radiation (air kerma) were measured at selected locations. Moreover, the radiology nurse operating the scanner and the primary clinician attending the examinations wore passive dosimeters on their protective glasses to estimate the eye lens dose (*H_p_*(3)) and active dosimeters on the chest pocket of their protective aprons to estimate the effective dose (*H_p_*(10)).

**Results:**

The integrated radiation shielding typically achieves a 91%-99% dose reduction such that, over a 3-month period, neither the ambient dose nor the eye lens doses measured exceeded the minimum detectable level of the dosimeters. The average doses measured by active personal dosimeters across 31 adult patient examinations were 0.07 ± 0.03 µSv for the operating nurse and 0.03 ± 0.02 µSv for the attending clinician.

**Conclusions:**

Mobile head CT with integrated shielding can substantially reduce occupational exposure and, with appropriate radiation safety training, obviate the need for additional personal protective equipment, especially when personnel remain behind the operator console.

**Advances in knowledge:**

This work provides practical guidance to ensure occupational radiation safety when using a mobile head CT system in the NICU.

## Introduction

Patients admitted to a neurointensive care unit (NICU) frequently require computed tomography (CT) scans, either urgently due to declining neurological function or as part of the routine preoperative and postoperative evaluation. Typically, this involves moving a critically ill patient from the NICU to a CT suite located in the radiology department. Given their critical health status, such transportation risks compromising patient safety and worsening clinical outcomes.^1^^,^^2^ A viable solution is to perform the scan within the NICU using a mobile head CT system.^3^^,^^4^

A mobile head CT system allows examinations to be performed directly at the point of care, potentially reducing the time interval between diagnosis and intervention. In addition to improving patient care, a mobile CT scanner can be a cost-effective investment, as it enables timely examinations for non-intensive care patients and reduces the expenses associated with transporting patients between departments.^5^ However, the implementation of a mobile CT warrants caution, as clinical staff, nearby patients, and relatives could be exposed to radiation.^6^

Several mobile head CT systems have been developed over the years, including the compact OmniTom and CereTom scanners from NeuroLogica and the xCAT IQ cone-beam CT (CBCT) system from Xoran Technologies. It is important to note that although earlier mobile CT systems offered inferior image quality relative to conventional stationary CT,^7^^,^^8^ the latest iterations have improved considerably.^9^ A prominent example is the newly developed SOMATOM On.site scanner from Siemens Healthineers. This scanner combines proven technologies from Siemens conventional CT scanners with a design for mobile use, resulting in image quality comparable to that of typical stationary scanners.^10^ It has a telescopic gantry that glides on the system trolley during scanning, and it comes with a foldable lead curtain to cover the front of the gantry opening, as well as a lead shield to cover its back.

The purpose of this study is to investigate radiation exposure in a NICU equipped with the On.site mobile head CT system, with a particular focus on strategies to reduce occupational exposure by implementing suitable radiation protection measures.

## Methods

### Mobile head CT

SOMATOM On.site is a compact CT system well-suited for imaging in the intensive care unit. It features a 35-cm retractable gantry that glides over the system trolley during scanning, and it comes with integrated neck and head support. Although it offers a limited range of scanning parameters compared to standard CT scanners, it provides the essential settings for head scans. The imaging protocol used in this work consists of typical brain imaging settings, albeit with automatic tube current modulation disabled, resulting in a CT dose index (CTDI_vol_, 16 cm) of 45 mGy. The scans were performed in helical mode at 120 kV tube voltage, with a pitch of 0.35, a beam collimation of 32 *×* 0.75 mm, and a 1.0 s rotation time.

**Figure 1:**
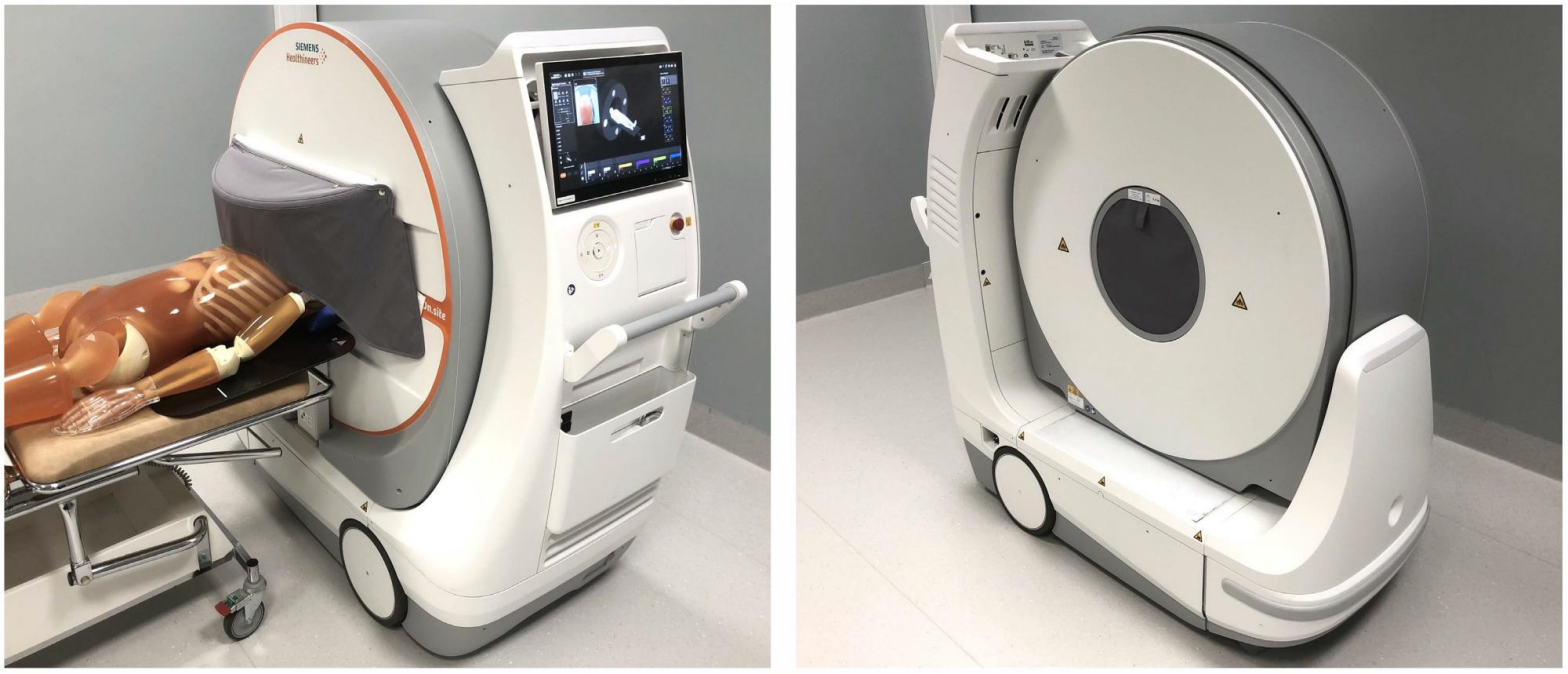
Siemens SOMATOM On.site mobile head CT system with a foldable 0.5-mm lead curtain covering the front of the gantry opening (photo on the left), and a detachable 1-mm lead shield covering its back (photo on the right). The photo on the left also includes the anthropomorphic whole-body phantom (PBU-60), positioned on a patient trolley to simulate scatter radiation.

Two key radiation protection features are included as standard with the On.site scanner. These are a foldable 0.5-mm lead curtain designed to cover the front of the gantry opening during scanning and a detachable 1-mm lead shield that covers its back (shown in Figure 1). Both components are manufactured by MAVIG GmbH and have been tested in accordance with the International Electrotechnical Commission (IEC) standard 61331-1.^11^ To ensure that the patient remains visible even with the front radiation cover closed, the system is fitted with bore lighting and a camera, whose live feed is displayed on the operator console. It should also be noted that the CT gantry itself is shielded. Together, these features are intended to enable the use of the scanner in hospital areas with limited shielding.

### Measuring the scatter radiation dose

**Figure 2:**
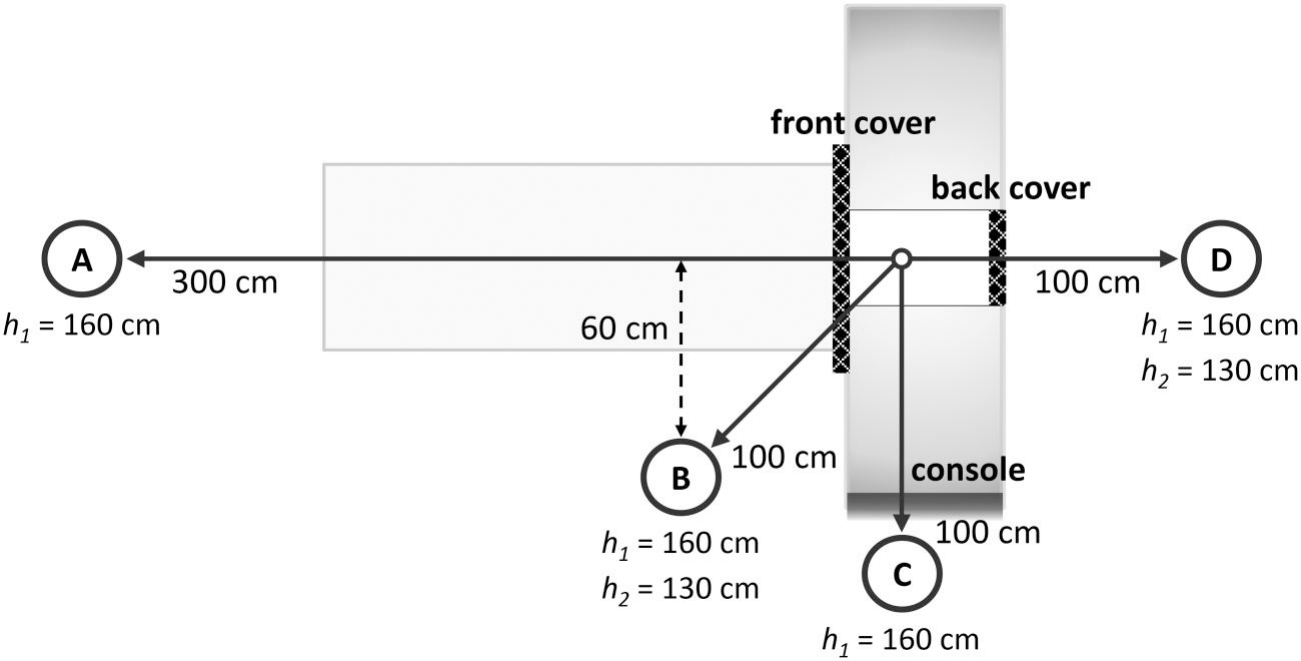
Schematic representation of the scatter radiation dose measurement positions, designated as (A-D), each illustrating a possible clinical scenario as discussed in the text. The positions represent 1 or 2 heights: 160 cm for a typical eye level and 130 cm for the chest. The front and back covers depict the placement of the protective lead shields integrated into the mobile head CT system.

The scatter radiation dose was determined using a large-volume (1800 cm^3^) cylindrical open-air ionization chamber (10X6-1800 Radcal) mounted on a tripod stand, with the height adjusted to represent either a typical torso or eye level. The instrument measures air kerma free-in-air, with calibration traceability to the Swedish secondary standard laboratory. The measurement positions were carefully selected to encompass several possible clinical scenarios, as illustrated in Figure 2:

1. Clinicians may observe the patient from a distance
2. A clinician may attend to the patient
3. An operator manages the CT scan from the operator console
4. A nurse might manage critical medical equipment behind the gantry

An anthropomorphic whole-body phantom (PBU-60, Kyoto Kagaku) was placed on a patient trolley to simulate the scatter radiation from a patient receiving a head CT scan (depicted in Figure 1). The phantom incorporates a synthetic skeleton embedded in soft tissue substitute, making it a suitable artificial body for the production of scatter radiation.

### Measuring the occupational dose

Occupational dose measurements were performed at the NICU at Karolinska University Hospital, Stockholm, Sweden. Three different operational dose quantities established by the International Commission on Radiation Units and Measurements (ICRU)^12^ were considered to fully characterize the occupational dose:

1. Ambient dose equivalent at 10 mm depth, *H*^∗^(*d*=10 mm), which serves as the operational quantity for estimating effective dose in area monitoring.
2. Personal dose equivalent at 3 mm depth, *H_p_*(*d*=3 mm), which serves as the operational quantity for estimating equivalent eye lens dose in staff monitoring.
3. Personal dose equivalent at 10 mm depth, *H_p_*(*d*=10 mm), which serves as the operational quantity for estimating effective dose in staff monitoring.

#### Ambient and eye lens dose

**Figure 3:**
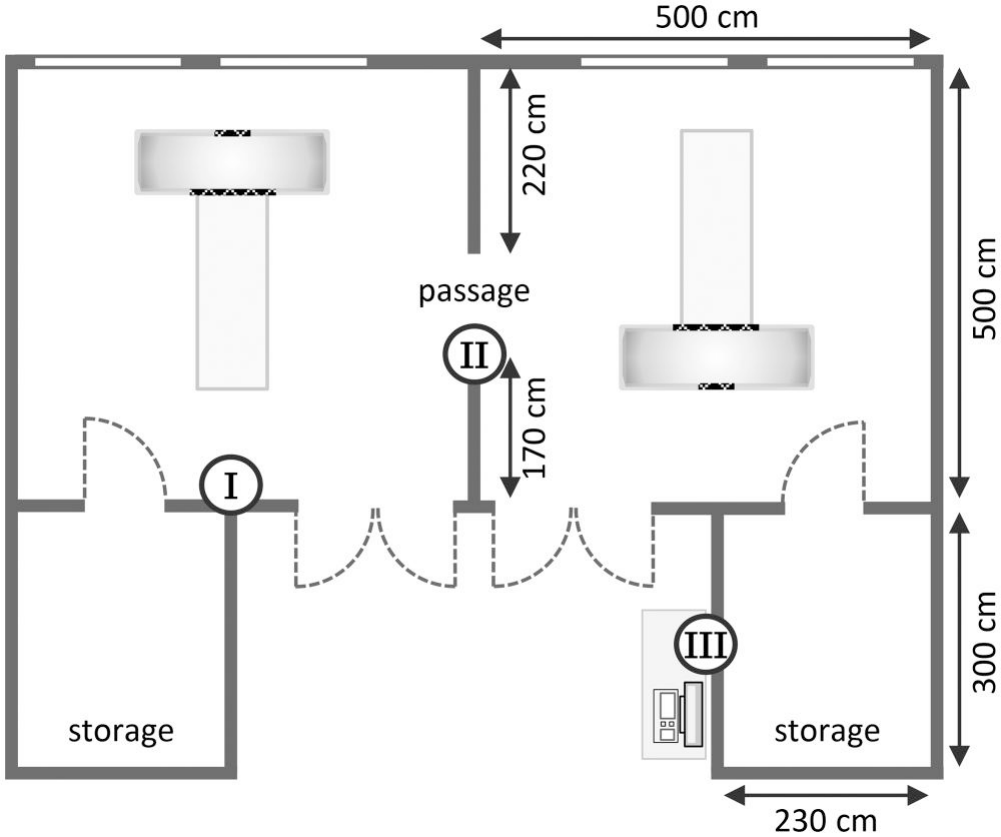
Schematic representation of the locations for ambient dose measurements in 1 section of the NICU (dosimeters were installed in 5 such sections). The measurement positions correspond to: (I) opposite the front of the gantry, (II) the passage between the 2 adjoining rooms, and (III) the workstation situated outside. Note that the mobile head CT system was oriented differently depending on the room used due to the mirrored arrangement of critical medical equipment.

The ambient dose equivalent was surveyed for 3 months using IPLUS optically stimulated luminescence (OSL) area dosimeters from LANDAUER with a minimum detectable level of 0.05 mSv. These dosimeters were mounted on the walls shown in Figure 3, at a height of 130 cm above the floor. Concurrently, the occupational eye lens dose was evaluated for 2 radiology nurses operating the mobile head CT system, who wore LANDAUER VISION thermoluminescence dosimeters (TLD) on both the right and left sides of their protective glasses. These dosimeters measure the personal dose equivalent *H_p_*(*d *= 3 mm) with a minimum detectable level of 0.1 mSv. Both types of LANDAUER dosimeters used in this study function as passive dosimeters, providing the cumulative dose over the monitoring period. The area dosimeters were returned to LANDAUER for readout after the 3-month survey, whereas the personal dosimeters were exchanged monthly.

#### Personal dose

The radiology nurse operating the mobile head CT system and the primary attending clinician wore RaySafe i3 personal dosimeters (Unfors RaySafe) on the chest pocket of their protective aprons during 31 adult patient examinations. These dosimeters measure the personal dose equivalent *H_p_*(*d *= 10 mm) with a 1 s integration time, are calibrated with traceability to the Physikalisch-Technische Bundesanstalt (PTB), and have a minimum detectable dose-rate of about 0.01 µSv/s.

The Raysafe i3 personal dosimeters operate as active dosimeters, recording the dose associated with each individual scan, thereby enabling a detailed analysis of staff exposure.^13–16^ They have improved performance relative to the earlier i2 system,^17^ which has been validated and found to perform comparably to passive dosimeters.^18^^,^^19^

### Statistical analysis

Descriptive summary statistics are reported as the mean with 1 SD or as relative difference, depending on the requirements of the specific analysis. Comparative analyses of the measured doses were performed using a paired sample t-test, with a significance level (alpha) of 0.01.

The scatter radiation doses measured with the ionization chamber were estimated to have a relative combined SD of 7%. This was determined by the propagation of uncertainty, considering the repeatability of the measurements, the alignment of the instrument (reproducibility), and the calibration uncertainty. The doses measured with active personal dosimeters were estimated to have a relative combined SD within 20%, according to the manufacturer’s specifications and adjusting for the clinical circumstances of this study. The factors are energy dependence, angle of radiation incidence (*<*15%), and dose rate (*<*10%).

## Results and discussion

### Scatter radiation dose

Table 1 presents the scatter radiation distribution from a standard head CT examination performed with the mobile head CT system. The findings show that the integrated radiation shielding provides a considerable dose reduction. The scatter radiation is reduced by between 91% and 99% (*P < *10^− 7^) in all directions, except behind the operator console (position C). This is attributable to the substantial shielding already provided by the gantry, which results in low exposure levels even without additional shielding. As a result, the safest location is, as anticipated, along the short side of the gantry, where the operator console is situated. By contrast, the least protected areas are beside the patient (position B) and behind the gantry (position D), although at position D, the top of the gantry helps to attenuate the scatter radiation dose at eye level. Regardless, in all positions, the integrated radiation shielding substantially reduces scatter radiation.

**Table 1:** Scatter radiation dose measured for a standard head CT examination, with (shielded) and without (unshielded) the integrated radiation shielding covering the gantry opening of the mobile head CT system.^a^

Pos.	Level	Unshielded (µGy)	Shielded (µGy)	Difference (%)
A	Eye	4.24	0.37	−91
B	Eye	35.34	1.55	−96
	Chest	63.18	3.15	−95
C	Eye	0.85	0.31	−64
D	Eye	15.32	0.56	−96
	Chest	84.32	0.58	−99

a The different measurement positions (A-D) are detailed in Figure 2. Note that the doses presented are mean values with a relative combined SD of 7%.

**Figure 4:**
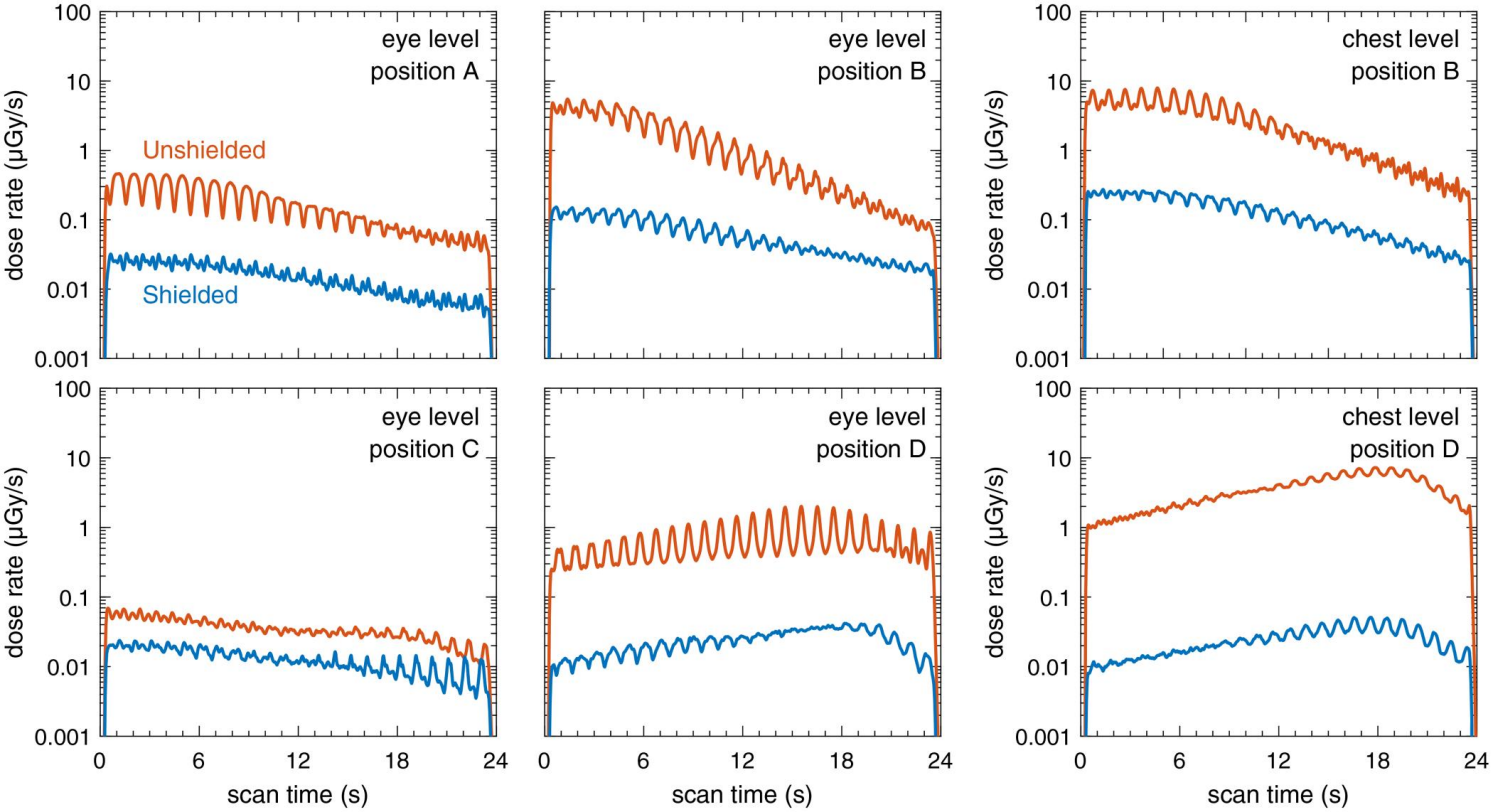
Scatter radiation dose rate as a function of time for a standard head CT examination, with (shielded) and without (unshielded) the integrated radiation shielding covering the gantry opening of the mobile head CT system. The different measurement positions (A-D) are detailed in Figure 2. Note that the dose rate is on a logarithmic scale and that the observed ripple is a characteristic of the helical acquisition mode.

Another aspect worth examining is how the scatter radiation dose changes throughout the duration of a scan. The scatter radiation dose rate as a function of scan time has thus been captured in Figure 4. It can be seen that on the side where the patient table is located (positions A, B, and even C), the dose rate decreases as the scan progresses, whereas the opposite trend occurs behind the gantry (position D). Recall that the gantry of the mobile head CT system glides during the examination to cover the length of the scan volume, unlike stationary CT, where the gantry remains fixed and the patient table moves through the gantry opening. Consequently, the distance between the scatter source and a given measurement point varies over the course of the scan for the mobile head CT system, which, in turn, impacts the scatter radiation dose rate accordingly. The scatter radiation dose per examination, therefore, does not scale linearly with scan length, rendering the commonly used metric of scatter radiation dose normalized by the dose-length product (DLP), which is the CT dose index (CTDI_vol_) times the scan length, ineffective for assessing staff exposure. For this reason, this metric has not been evaluated in this study; instead, we refer to Figure 4 to understand how the dose changes with different scan lengths, recognizing that each second represents a scan length of 0.84 cm (as inferred from the known scan length and scan duration).

### Ambient and eye lens dose

None of the area dosimeters installed in the NICU registered doses that exceeded their minimum detectable level, meaning that the ambient dose equivalent remained below 0.05 mSv. The eye lens dosimeters worn by the staff showed a similar result, as none of the measured doses exceeded the minimum detectable level of 0.1 mSv. It must be stressed, however, that these results are contingent on the clinical workload during the study period. At present, the mobile head CT is used for fewer than 3-4 examinations per week. This low examination rate limits the usefulness of exposure estimates based solely on passive dosimeters with a relatively high minimum detectable level. To address this, the next section provides an analysis based on active dosimeters that have measured the personal dose for each examination.

### Personal dose

**Figure 5:**
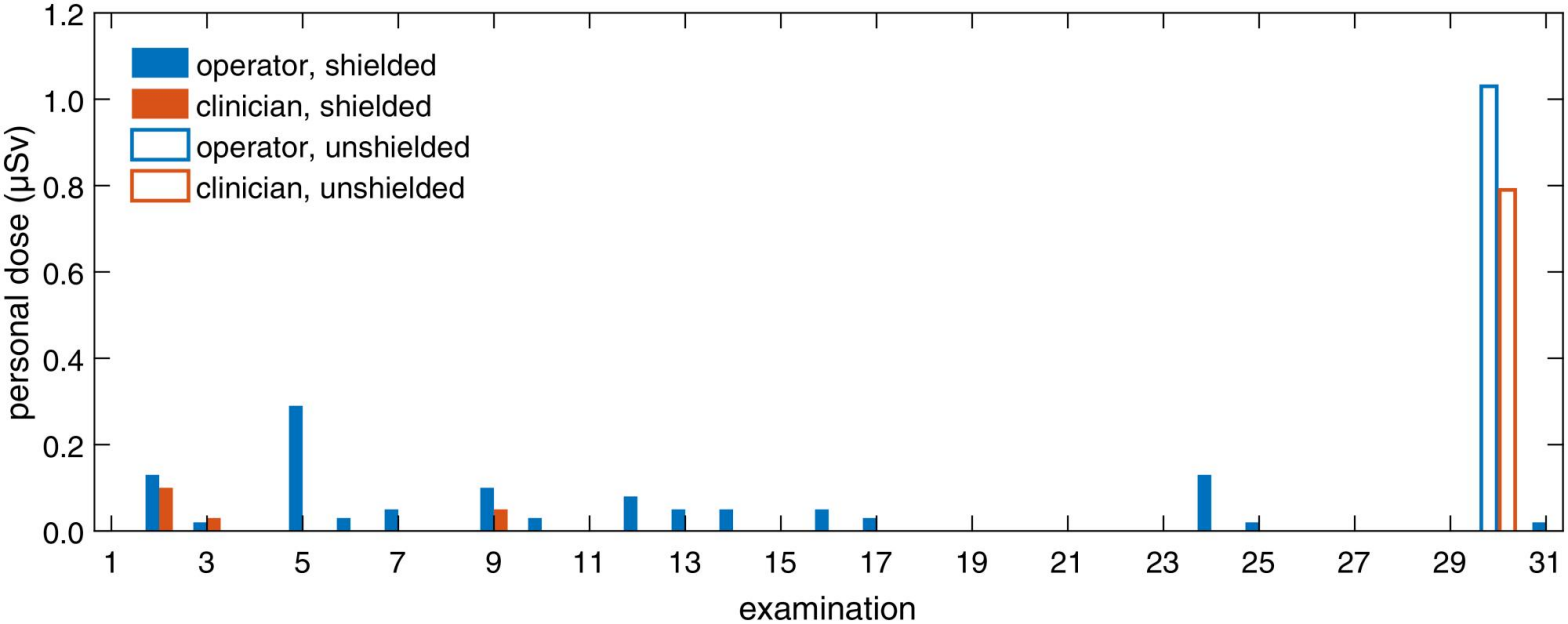
Doses measured by personal dosimeters worn at chest level by the radiology nurse operating the mobile head CT system (operator) and the primary clinician attending the examination (clinician). The terms “shielded” and “unshielded” indicate, respectively, whether the integrated radiation shielding was in use or not. Note that apparent missing values represent measurements below the minimum detectable dose rate level.

Figure 5 shows the occupational doses measured using active personal dosimeters worn by the clinical staff during each head CT examination performed with the mobile head CT system. The measurements show an average staff dose per examination of 0.07 *±* 0.03 µSv for the radiology nurse operating the scanner and 0.03 *±* 0.02 µSv for the primary clinician attending the examination. The maximum recorded dose of approximately 1 µSv occurred on the only occasion when the integrated radiation shielding was not employed by the staff.

The findings indicate that staff doses can be kept well below both the occupational exposure limit of 20 mSv effective dose and the public exposure limit of 1 mSv effective dose.^20^ It is worth noting that, even on the one occasion when the integrated radiation shielding was not employed, the recorded exposure was still limited. This is largely due to the deliberate positioning of personnel during the scan. As corroborated by the scatter radiation dose measurements (see Table 1), the radiology nurse operating the scanner can substantially lower their exposure by standing directly behind the operator console (denoted as position C in Figure 2), while the primary clinician attending the examination should similarly remain behind the console or stay at a considerable distance from the gantry during the scan (position A in Figure 2). It should also be mentioned that, in this study, the primary clinician typically chose to leave the room while the scan was being performed, which accounts for their generally lower exposure compared to the operator.

The low exposure measured with the integrated radiation shielding covering the gantry opening suggests that, under certain conditions, head CT examinations in the NICU can be performed without requiring personal protective equipment such as aprons. This not only improves staff comfort but also demonstrates that the mobile head CT system can be operated safely in hospital areas with limited radiation shielding. Indeed, based on the measurements in this study, the clinical staff would have to be involved in more than 1000 mobile head CT examinations to reach a cumulative effective dose of only 0.1 mSv. Without the use of the integrated radiation shielding, this threshold would be reached after approximately 100 examinations. These projections, of course, assume consistent practice patterns and that basic radiation safety precautions are routinely applied to minimize exposure, such as avoiding proximity to the gantry unless it is necessary.

In order to ensure safe radiation protection practices, it is essential that clinical staff who operate the mobile head CT system receive appropriate radiation safety training. This training should include proper techniques for positioning the frontal lead curtain on the patient, securing the back cover, and reiterating the essential radiation protection principles of time, distance, and shielding.^21^ Furthermore, when clinical circumstances prevent the use of the integrated radiation shielding, personal protective equipment (such as aprons and leaded eyewear) should be considered to keep radiation exposure as low as reasonably achievable.

## Conclusions

This study demonstrates that a mobile head CT system equipped with integrated radiation shielding can substantially reduce occupational radiation exposure. When combined with appropriate radiation safety training, including correct positioning of the frontal lead curtain and securing the back cover, the integrated shielding can obviate the need for additional personal protective equipment. This is particularly the case when staff can remain directly behind the operator console or maintain sufficient distance from the gantry.
